# Robotic Aortic Annular Enlargement With Y-Incision and Rectangular Patch

**DOI:** 10.1177/15569845251375582

**Published:** 2025-09-16

**Authors:** Ahsan Ehtesham, Jai Parkash, Muhammad Zain Shaikh, Korey Zellner, Ghulam Murtaza

**Affiliations:** 1 Hamad Medical Corporation, Doha, Qatar; 2 SSM Health Heart & Vascular Care Center, Madison, WI, USA; 3 Liaquat University of Medical and Health Sciences, Jamshoro, Pakistan; 4 University of Wisconsin Northern Illinois, Rockford, IL, USA

Surgical enlargement of the aortic annulus enables insertion of a larger prosthetic valve, avoiding patient–prosthesis mismatch (PPM).^1^ Although several aortic annular enlargement techniques have been described, the rectangular patch technique with an inverted Y-incision along the left and noncoronary cusps is relatively recent.^2,3^ These procedures are technically challenging via minimally invasive approaches, but robotic assistance makes them possible.

The patient had symptomatic severe aortic valve stenosis with evidence of left ventricular dysfunction (ejection fraction = 45%, peak gradient = 47.2 mm Hg, mean gradient = 29.3 mm Hg, and aortic valve area = 0.6 cm^2^). Preoperative computed tomography measured the aortic annulus at 18 mm (patient body surface area = 1.9). Notably, moderate aortic stenosis was documented 6 months earlier with preserved ejection fraction (55% to 65%), indicating rapid disease progression.

During the surgery, cannulation of the right groin vessels was performed to establish cardiopulmonary bypass. Simultaneously, the thoracic cavity was entered through a minithoracotomy incision in the anterior axillary line at the level of the fourth intercostal space.^4^ Three 8 mm robotic ports were inserted surrounding the access incision. Dense pericardial adhesions were taken down to expose the aorta and the left interatrial groove. An antegrade cardioplegia catheter was placed in the distal ascending aorta, and a left ventricular vent was placed via right superior pulmonary vein. The robot was docked with DeBakey forceps, long bipolar forceps, and monopolar scissors in arms 1, 3, and 4, respectively (Supplemental Fig. 1). The camera was introduced through the access port. The aorta was cross-clamped using a flexible clamp Cygnet® Flexible Clamp (Peters Surgical, Boulogne-Billancourt, France), and the heart was arrested using antegrade del Nido cardioplegia.

An oblique aortotomy was made, revealing a severely diseased bicuspid aortic valve. Following complete excision of the valve and thorough debridement of the annulus, measurement revealed a diminutive annular diameter of barely 19 mm, which was inadequate according to the recommendation of the prosthetic valve manufacturer (Edwards Lifesciences, Irvine, CA, USA). To address this limitation, the aortotomy was extended along the commissure between the left and noncoronary cusps, continuing to the nadir of both sinuses in an inverted Y-shaped configuration. A 3 cm wide bovine pericardial patch was then sutured into position, beginning at the nadir of the left coronary cusp, anchored inferiorly to the aortomitral curtain, and laterally sewn up the ascending aortic wall.

This enlargement procedure enabled the successful implantation of a 23 mm bioprosthesic valve. After stitching the annular valve sutures through the sewing ring of the prosthesis and after properly seating the valve, the sutures were tied with the COR-KNOT® device (LSI SOLUTIONS®, Victor, NY, USA). The aortotomy was then closed, and the patient was gradually weaned from cardiopulmonary bypass. Intraoperative transesophageal echocardiography confirmed excellent prosthetic valve function with no paravalvular leak and a mean gradient of 4 mm Hg (Supplemental Fig. 2).

The patient’s postoperative course demonstrated steady improvement, allowing transfer to the regular cardiac surgery ward and discharge to home on the seventh postoperative day. Her 2-week outpatient follow-up evaluation showed marked improvement in her symptoms.

PPM occurs when the prosthetic valve’s effective orifice area is inadequate for the patient’s body surface area. The Y-incision/rectangular patch technique is preferred for aortic annular enlargement due to its simplicity and effectiveness,^3^ enabling the placement of a larger, better suited prosthesis, ensuring adequate valve-to-coronary distance for potential future valve-in-valve transcatheter aortic valve replacement procedures while restoring normal aortic valve hemodynamics.

This case represents one of the first reported robot-assisted aortic annular enlargements, demonstrating the expanding potential of robotic cardiac surgery (Supplemental Video).

## Supplemental Material

sj-docx-1-inv-10.1177_15569845251375582 – Supplemental material for Robotic Aortic Annular Enlargement With Y-Incision and Rectangular Patch Supplemental material, sj-docx-1-inv-10.1177_15569845251375582 for Robotic Aortic Annular Enlargement With Y-Incision and Rectangular Patch by Ahsan Ehtesham, Jai Parkash, Muhammad Zain Shaikh, Korey Zellner and Ghulam Murtaza in Innovations
