# Supplementary material for: Robotic Aortic Annular Enlargement With Y-Incision and Rectangular Patch
Source: Innovations (Phila). 2025 Sep 16;20(5):497–8. doi: 10.1177/15569845251375582 (PMC12575811; doi:10.1177/15569845251375582)
Supplement: sj-docx-1-inv-10.1177_15569845251375582 – Supplemental material for Robotic Aortic Annular Enlargement With Y-Incision and Rectangular Patch [file sj-docx-1-inv-10.1177_15569845251375582.docx]

**Supplemental Figure Legends**

**Supplemental Fig. 1.** Setup of the operation showing the minithoracotomy and placement of the robotic arms.

**Supplemental Fig. 2.** Post-bypass TEE image showing the gradient across the valve.

**Video Legend**

**Supplemental Video.** Narrated case summary including relevant preoperative details and surgical technique.
